# Three dimensional finite element analysis used to study the influence of the stress and strain of the operative and adjacent segments through different foraminnoplasty technique in the PELD: Study protocol clinical trial (SPIRIT Compliant)

**DOI:** 10.1097/MD.0000000000019670

**Published:** 2020-04-10

**Authors:** YiZhou Xie, Xinling Wang, Qiang Jian, Xiaohong Fan, Yang Yu, Dangwei Gu, WeiDong Wu

**Affiliations:** a Chengdu University of Traditional Chinese Medicine; b Hospital of Chengdu University of Traditional Chinese Medicine, Chengdu, Sichuan Province; c Southern Medical University, Guangzhou, Guangdong Province, P.R. China.

**Keywords:** biomechanical, foraminoplasty, percutaneous endoscopic lumbar disectomy, 3-dimensional finite element model

## Abstract

**Introduction:**

Percutaneous endoscopic lumbar disectomy (PELD) is one of the most popular minimally invasive techniques of spinal surgery in recent years. At present, there are 2 main surgical approaches in PELD: foraminal approach and interlaminar approach. What's more, foraminoplasty is a necessary step for both approaches. However, there are few biomechanical studies on the formation of different parts of the intervertebral foramen. The aim of this study is to explore the effects of different foraminoplasty methods on the biomechanics of the corresponding and adjacent segments of the lumbar through a 3-dimensional finite element model analysis.

**Methods:**

We established a normal 3-dimensional finite element mode of L3 to L5, simulated lumbar percutaneous endoscopy by doing cylindrical excision of bone whose diameter was 7.5 mm on the L5 superior articular process and the L4 inferior articular process, respectively, so that we obtained 3 models: the first one was normal lumbar model, the second one was the L4 inferior articular process shaped model, and the third one was the L5 superior articular process shaped model. We compared the biomechanics of the intervertebral disc of L3/4 and L4/5 when they were in the states of forward flexion, backward extension, left and right flexion, and left and right rotation on specific loading condition.

**Discussion:**

If the outcomes indicate the trial is feasible and there is evidence that one of the foraminoplasty technique may make few differences in biomechanics of corresponding lumbar intervertebral disc, we will proceed to a definitive trial to test the best way to foraminplasty, which could make biomechanical influence as little as possible.

Trial Registration: Chinese Clinical Trial Registry, ChiCTR1900026973. Registered on September 27, 2019.

## 1 Introduction

Lumbar disc herniation is a kind of disease with a series of clinical manifestations caused by degeneration of intervertebral disc tissue because of various reasons, which results in compression of dural sac or nerve root in posterior spinal canal.^1^,^2^,^3^ With the aging of the social population structure, the incidence of this kind of disease is increasing, seriously affecting the quality of patients’ daily life. In recent years, due to the rapid development of percutaneous endoscopic lumbar disectomy (PELD), it has the advantages of small incision, less tissue trauma, less bleeding, fast recovery, early movement, fewer complications, relatively low operation cost, and marked reduction of wound infection rate compared with the traditional open surgery when facing some diseases such as lumbar disc herniation and stenosis of lumbar spinal canal.^4^,^5^,^6^,^7^,^8^ Although foraminoplasty is widely adopted as a key technique in PELD, there is no careful research and conclusive evidence on the influence of the stress (disc, facet joint) and strain (range of motion in different directions) of the corresponding segment or adjacent segment after foraminoplasty with different location and size.^9^,^10^

In this study, 3-dimensional finite element method is selected to analyze the stress and strain of the above-mentioned situations, which can exclude the influence of other factors. The results of calculation have strong repeatability and avoid the difficulty of corpse specimen sampling. It has unique advantages in simulating human body structure and analyzing biomechanical characteristics.

Throughout the research on foraminoplasty of PELD worldwide, most of them are limited to the analysis on the clinical efficacy of different types of foraminoplasty, but few researches have been reported on the biomechanical research which has far-reaching impact on the mid-and long-term spinal degeneration after surgery. In order to simulate the different forming on intervertebral foramen, the 3-dimensional finite element method was used to simulate the osteotomy of different parts (tip, waist, basal) and sizes (diameter 7.5, 10, 12 mm) on the L5 superior articular process in L4/5 segment of PELD, so as to study the influence of stress and strain for corresponding segment (L4/5) and adjacent segments (L3/4, L5/S1) through different methods of foraminoplasty.

## 2 Objective

A reasonable and effective 3-dimensional finite element model of L3 to S1 of the lower lumbar spine was constructed by 3-dimensional finite element method, and on the basis of the model, 3-dimensional finite element models of L4/5 with different foramen forming modes were constructed. By analyzing the stress and strain of the above models under different motion states, we could know:

- which region of foramina formation has less influence on the mechanics of the intervertebral disc or facet joint of corresponding segment or adjacent segment and on the stability of the spinal segment?
- what diameter of foramina formation has less influence on the mechanical properties and stability of the spinal segment?

It is small and convenient for clinical operation.

## 3 Methods

### 3.1 CT data source

The CT images of the L3 to L5 of 40 male and 40 female normal adults in China were collected. The trial was examined and approved by the Ethics Committee of the Hospital of Chengdu University of traditional Chinese medicine (No. NS-5279).

### 3.2 Applicants inclusion criteria

The age of applicants is between 20 and 60 years old in good health and they are good at spirit and intelligence. They obey the arrangement of the research group, accept the treatment plan designed by the research group and sign the informed consent.

### 3.3 Applicants exclusion criteria

Participants suffered from severe spinal degeneration or severe irreversible damage of multiple spinal columns such as spinal tuberculosis and tumor.

### 3.4 Relevant software and equipment

Siemens Somatom Sensation 64-slice spiral CT is used for CT scanning; surface design software Creo 3.0, medical image processing software Mimics 16.0, and reverse engineering software Geomagic Studio 12.0 are used for 3-dimensional model building; finite element analysis software ANSYS 15.0 is used for 3-dimensional model processing and biomechanical finite element analysis.

### 3.5 Establishment of L3 to S1 normal 3-dimensional finite element model

We used spiral CT to scan L3-S1 and save the 2-dimensional cross-sectional images in DICOM format, then input the DICOM files into Mimics 16.0 software to establish the 3-dimensional geometric model of L3-S1 in Mimics software, and to import the model into ANSYS 15.0 software after polishing and smoothing for meshing, so as to make the 3-dimensional finite element model. According to the anatomical position of each ligament, the normal 3-dimensional finite element model of L3 to L5 was established by adding the intervertebral disc, anterior longitudinal ligament, posterior longitudinal ligament, yellow ligament, interspinous ligament, supraspinal ligament, and intertransverse ligament.^[11]^ According to the references, the structure of the model was assigned on the basis of the normal tissue parameters.^12^,^13^ Its starting and ending points and cross-sectional area imitate the anatomical observation conclusions of the relevant segments as far as possible. What's more, the contact relationship and friction coefficient of the articular surface are defined at 0.1.^[14]^

### 3.6 Validation of normal model

The above-mentioned normal finite element model was applied with the same constraints and loads as the research on cadaver specimens in the literature, and the mobility in different directions was compared.^[15]^ After repeated revision of the relevant ligament structure, the mobility of each spinal segment (L3/4, L4/5, L5/S1) in the 6 directions of flexion, extension, lateral flexion, and left-right rotation were distributed in the biomechanical experiment of cadaver specimens. Within the scope, the model is validated to be effective and reliable.

### 3.7 Establishment of foraminoplasty models in different parts and sizes

**Figure 1:**
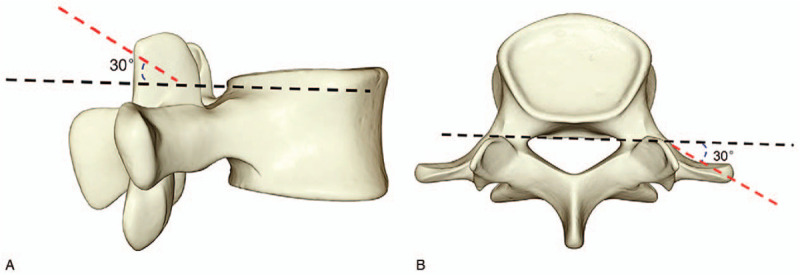
The direction of foraminoplasty.

**Figure 2:**
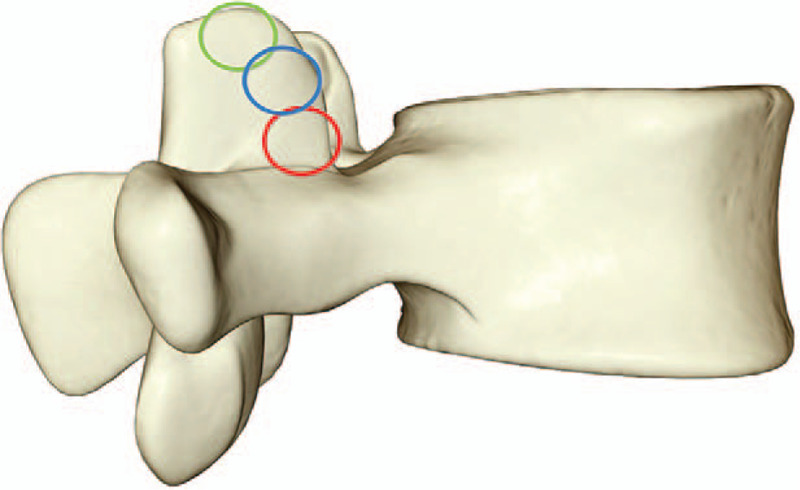
Different parts of foraminoplasty.

On the basis of the above L3 to S1 normal model which has passed the validation, the percutaneous endoscopic technique of lumbar spine is simulated. The tip, waist and base of L5 superior articular process were used as puncture points respectively to establish the precise puncture guidance route. The tail inclined 30° to the cephalic side and formed an angle of 30° with the coronal surface (see Fig. 1). The right articular process tip (green area) was excised along the direction of the route, waist (blue area) and base (red area) (the diameter of resection is 7.5 mm) to obtain the different parts of the articular process forming model (see Fig. 2).

**Figure 3:**
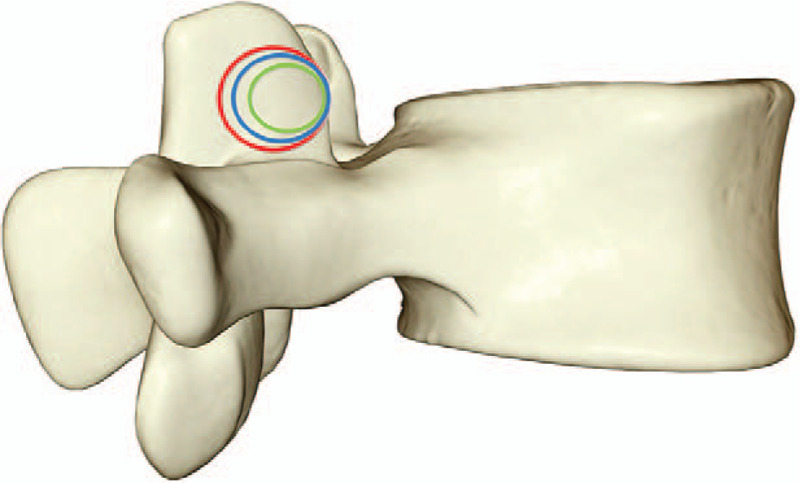
Different sizes of foraminoplasty.

With the central and ventral side of L5 superior articular process as the puncture point, the same puncture guide route was established. Bone of different sizes [7.5 mm (green area), 10 mm (blue area), and 12 mm (blue area)] were removed along the guide line, and the different sizes of the articular process models were obtained (see Fig. 3).

### 3.8 Loading of post-operative model

The inferior surface of the 3-dimensional finite element model with foraminoplasty was restrained by degrees of freedom. Different vertical loads were applied on the superior surface of L3 centrum to simulate the standing or sitting position of normal human body. The same pure torque was applied in the directions of flexion, extension, left and right bending, and left and right rotation, respectively, as in cadaveric specimens, with flexion, extension, left and right bending, and left and right rotation. Six kinds of motion states are loaded.

### 3.9 Three-dimensional finite element analysis of the model

The stress and strain of normal model, 3 different parts of L5 superior articular process (tip, waist, base) and 3 different sizes of L5 superior articular process (7.5, 10, 12 mm) were analyzed and compared. Stress analysis (stress changes of this segment, adjacent segment intervertebral disc and bilateral small joints), strain analysis (changes of spinal mobility of this segment and adjacent segment). The entire procedure is shown in Table 1.

**Table 1:**
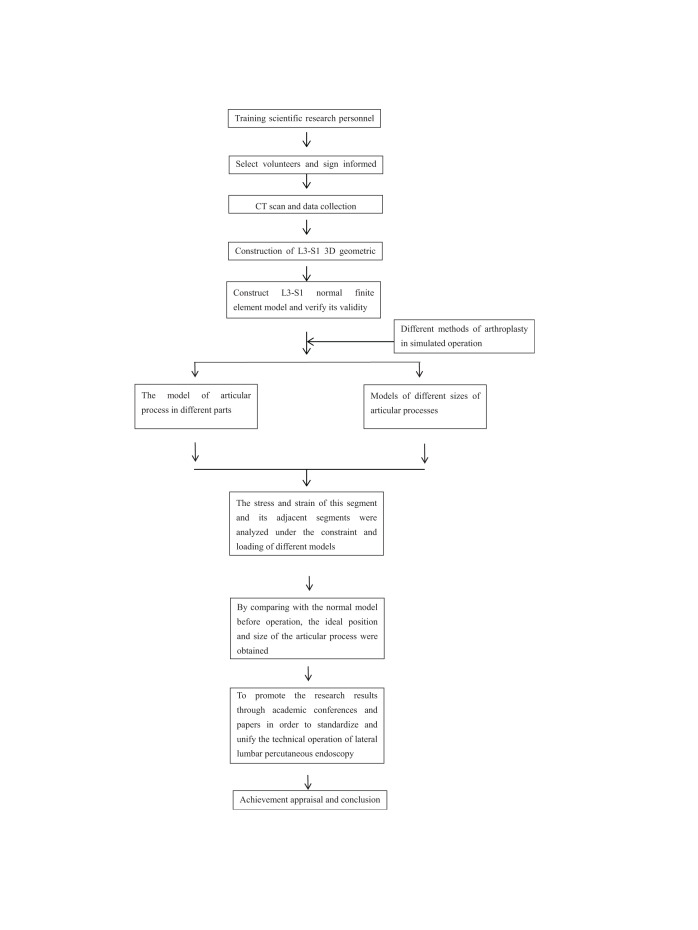
The entire procedure of the study.

### 3.10 Key scientific issues to be addressed

How to simulate the actual clinical state as real as possible with finite element model?

Firstly, CT data collection should be as complete as possible, and thin CT scanning should be used to avoid data loss. The rough points on the geometric model reconstructed by CT should be treated as smoothly as possible. After importing the 3-dimensional geometric model into ANSYS software, the appropriate element type is selected for multi-grid generation as much as possible, which is convenient for later calculation. Reconstruction of ligament structure, articular capsule, articular surface, and intervertebral disc is the key to high simulation of the model. The starting and ending points, cross-sectional area, and articular capsule reconstruction of the ligament structure should be as close as possible to the anatomical position, and the accuracy should be ensured by repeated adjustments after loading. The definition of the release relationship of articular surface and friction coefficient should be defined by reference to classical literature. For sliding contact, the friction coefficient is 0.1. The reconstruction of intervertebral disc structure should include fibrosis, nucleus pulposus, cartilage endplate, and other anatomical structures. Each structure should be endowed with different material properties according to its characteristics, and ultimately validated.

To simulate the operation of intervertebral foraminoplasty, different parts of the superior articular process of L5 were used as puncture points, and precise puncture guidance route was established. Cylindrical excision of the superior articular process of L5 was performed at the angle of 30° to coronal plane, respectively, to simulate the circumferential sawing of the articular process during the operation. According to the common position of the articular process and the size of the circular sawing, the resection site was defined. The resection diameters were 7.5, 10, and 12 mm for the tip, waist, and basal parts of L5 superior articular process, respectively.

Knowing the best position and size of articular process under the premise of convenient for clinical operation.

In the clinical operation of lumbar percutaneous endoscopy, the location and size of foraminoplasty vary from doctor to doctor or academic school, and there is no gold standard for operation. Doctors tend to pay more attention to the thoroughness of decompression or the convenience of manipulation, but they do not pay much attention to the location or size of the facet formation, and there will be less research on the impact of lumbar biomechanics in the future.

Through this study, we can determine which or which sites of foraminoplasty have less influence on the biomechanics and mobility of this segment or adjacent segments. The size of foraminoplasty is not only easy to operate, but also has the minimal impact on the structure of the spine. The clarity of these problems is of great significance for the standardization and unification of lateral lumbar percutaneous endoscopy technique in the future, so as to facilitate the popularization and development of this technique for the benefit of more patients.

### 3.11 Data management

The investigators and research assistants in the study team will collect the data and enter them directly into the electronic-Case Record Form. The data will be securely stored, with specific access rights granted to members of the study team according to their role in the study. Participants, healthcare professionals, the public, and other relevant groups could get the results of the trial as soon as we complete the trial and publish our achievements.

### 3.12 Safety

Any observed side effects related to radiation exposure will be documented throughout the trial period and reported to the sponsor without delay. These data will be provided for periodical review by the Data and Safety Monitoring Board.

## 4 Discussion

Lumbar is an important part of human activity and stress-bearing. It has deep location, complex anatomical structure, and adjacent relationship, and has important physiological functions. The mechanical research of this area has been an important subject in orthopedics field. The finite element method studies the stress and strain of the relevant region by meshing, constraining, and loading the model. This technology has been applied in medical field since 1960s. With the rapid development of computer technology, it has been applied more and more widely, and the simulation degree is getting higher and higher.^16^,^17^,^18^,^19^ The 3-dimensional finite element analysis method is a research method which established the relevant model and analyzed its stress and strain by the computer. The basic steps of its analysis are: creating model → defining material characteristics → gridding → setting load and constraint conditions → solving → result analysis. The 3-dimensional finite element model can simulate various tissues of human body, excluding the influence of other factors, and the calculation results have strong repeatability. In the aspect of simulating human body structure and analyzing biomechanical properties, it is incomparable with other methods.^20^,^21^,^22^ The application of finite element method in the study of lumbar biomechanics mainly includes the establishment of models, the biomechanical analysis of anatomical structures and implants. The displacement, stress and the corresponding relationship of any node in the model can be studied by using finite element analysis method.

However, the finite element model has made many simplifications and assumptions in the process of modeling, so the extent to which the established model can be simulated is a problem that researchers must consider. In order to ensure the accuracy of the follow-up study, the validity of the finite element model must be verified before further application.^14^,^23^,^24^ At present, the main verification method is to compare the results of the model test with those of the previous biomechanical test of cadaveric specimens. After the establishment of the finite element model, the model is loaded and the stress and strain of the model under flexion, extension, lateral bending, and rotating loads are observed respectively. The results of the test are compared with those of the previous relevant literature. If the 2 results are consistent, the model is valid. It can be applied for further research.

Since Professor Hooggland of Germany put forward the technique of “foraminoplasty” in PELD in 2002, percutaneous endoscopy of lumbar has made rapid progress in recent decades. Because of the improvement of doctors’ operation technology and the advancement of surgical instruments, the indications of operation are becoming more and more widespread. With the continuous development of this technology, different technical schools emerge at the historic moment, and there is often a lack of uniform or relatively standardized operating standards in the clinical operation process. Clinicians often pay more attention to how to achieve more thorough decompression, as well as the convenience and safety of operation, while less attention is paid to the trauma caused by technology itself or the biomechanics of spine after technology. Therefore, we often do not know the trend of spinal degeneration and the long-term clinical manifestations while achieving better short-term clinical efficacy.^[7]^

At present, in spinal surgery, although the research of lumbar finite element analysis has been widely and deeply carried out, it mainly focuses on:

1. stress and strain analysis of different internal fixation devices, to find the area of stress concentration for improvement or better prevention of failure of internal fixation devices
2. the biomechanical effects of different internal fixation methods were analyzed in order to select more suitable internal fixation methods^17^,^18^

Few studies have focused on changes in stress and mobility of the disc and facet joints at or near the lumbar segment after foraminoplasty at different sites or foraminoplasty of different sizes during percutaneous endoscopic lumbar surgery.

This trial will take L4/5 segment as an example to study the effects of foraminoplasty on the stress changes of L4/5 and adjacent L3/4, L5/S1 intervertebral discs, facet joints, and the motion changes of each segment in different regions of L5 superior articular process (tip, waist, basal) and sizes (7.5, 10, 12 mm in diameter), so as to provide a reference for lateral lumbar percutaneous endoscopy. It can provide detailed and exact biomechanical evidence and supply corresponding mechanical basis for further standardization of its operation, so as to make the technology better benefit the vast number of patients.

## Author contributions

Yizhou Xie, Wang Xinling, Yang Yu, Xiaohong Fan, and Weidong Wu performed the experiments. Yizhou Xie, Xinling Wang, and Yang Yu wrote the paper. Yizhou Xie, Yang Yu, Xiaohong Fan, Dangwei Gu, Weidong Wu, and Qiang Jian reviewed and edited the manuscript. All authors read and approved the manuscript.

YY and XFcontributed equally to this work and should considered as corresponding authors.
